# High-Throughput Sequencing Discloses the Cucumber Mosaic Virus (CMV) Diversity in Slovakia and Reveals New Hosts of CMV from the *Papaveraceae* Family

**DOI:** 10.3390/plants11131665

**Published:** 2022-06-23

**Authors:** Michaela Mrkvová, Richard Hančinský, Lukáš Predajňa, Peter Alaxin, Adam Achs, Jana Tomašechová, Katarína Šoltys, Daniel Mihálik, Antonio Olmos, Ana Belén Ruiz-García, Miroslav Glasa

**Affiliations:** 1 Faculty of Natural Sciences, University of Ss. Cyril and Methodius, Nám. J. Herdu 2, 91701 Trnava, Slovakia; michaela.mrkvova@ucm.sk (M.M.); hancinsky1@ucm.sk (R.H.); Peter.Alaxin@savba.sk (P.A.); Jana.Tomasechova@savba.sk (J.T.); daniel.mihalik@ucm.sk (D.M.); 2 National Agricultural and Food Centre, Research Institute of Plant Production, Bratislavská cesta 122, 92168 Piešt’any, Slovakia; 3 Biomedical Research Center of the Slovak Academy of Sciences, Institute of Virology, Dúbravská cesta 9, 84505 Bratislava, Slovakia; Lukas.Predajna@savba.sk (L.P.); Adam.Achs@savba.sk (A.A.); 4 Department of Microbiology and Virology, Comenius University in Bratislava, Ilkovičova 6, 84104 Bratislava, Slovakia; katarina.soltys@gmail.com; 5 Centro de Protección Vegetal y Biotecnología, Instituto Valenciano de Investigaciones Agrarias (IVIA), Ctra, Moncada-Náquera Km 4.5, 46113 Moncada, Spain; aolmos@ivia.es (A.O.); ana.belen.ruiz@uv.es (A.B.R.-G.)

**Keywords:** CMV, cucumovirus, genetic diversity, genome, high-throughput sequencing, satellite RNA

## Abstract

Cucumber mosaic virus (CMV; *Cucumovirus, Bromoviridae*) is an omnipresent virus characterized by a large host range and high genetic variability. Using high-throughput sequencing, we have characterized near complete genomes of 14 Slovak CMV variants from different plant hosts. Of these, three variants originated from the *Papaveraceae* species (oilseed poppy, common poppy and great celandine), previously poorly described as CMV natural hosts. Based on a BLAST search and phylogenetic analysis, the Slovak CMV isolates can be divided into two genetically different Groups, Ia and II, respectively. The SL50V variant, characterized by a divergent RNA2 sequence, potentially represents a reassortant variant. In four samples (T101, SL50V, CP2, MVU2-21), the presence of satellite CMV RNA was identified along with CMV. Although mechanically transmitted to experimental cucumber plants, the role of satellite RNA in the symptomatology observed could not be established due to a complex infection of original hosts with different viruses.

## 1. Introduction

Plant viruses pose a great threat to plant production worldwide. Due to the ongoing globalization of markets and highly efficient means of transportation of goods, plant viruses can reach new areas with potential for infecting new hosts in different environments more efficiently [1]. Generalist viruses, with wide host ranges, can be especially dangerous, as potential hosts are more frequently present in the environment [2].

Cucumber mosaic virus (CMV), the type species of the *Cucumovirus* genus (*Bromoviridae*), was first described more than a century ago [3,4]. CMV virion particles are isometric, with sizes of approximately 28–30 nm [5]. CMV has a tripartite genome that consists of positive-sense single-stranded RNAs packaged in separate particles [6]. Individual RNAs all have 5′ cap and 3′ tRNA-like structures. In addition to three genomic RNAs (RNA1, RNA2 and RNA3), two sub-genomic RNAs (RNA4 and RNA4a) contain protein-encoding open reading frames (ORFs). RNA 1 encompasses 1a ORF which contains methyltransferase and helicase domains. RNA 2 encodes ORF 2a containing RNA-dependent RNA polymerase domain, which together with 1a protein acts as a viral replicase. ORF 2b is translated from sub-genomic RNA4a, which is co-terminal with the 3′end of RNA2 and the 2b protein and acts as a viral suppressor of RNA silencing [7]. RNA 3 encodes ORF 3a, which corresponds to the movement protein (MP). ORF 3b corresponds to the coating protein (CP), which is translated from sub-genomic RNA4 co-terminal with the 3′ end of RNA3 [5,8]

Based on present knowledge, CMV isolates are classified into two major subgroups, termed I and II, according to their serological properties, symptomatology and CP sequence phylogenesis [9,10]. These groups show nucleotide sequence identities of 69% to 77% between groups, 88% identity within group I and 96% identity within group II [8]. Subgroup I is further divided into subgroup IA and IB based on RNA3 variability of the 5′ terminal noncoding region [10,11]. Subgroup IB is predominant in East Asia, while subgroups IA and II are commonly found all over the world [12]. It was previously noted that subgroup I isolates have greater high-temperature tolerance and are more prevalent in warmer climates and also that their symptoms are normally more severe when compared to subgroup II [13]. As an RNA virus with a divided genome, recombination and reassortment of viral strains are responsible for the genetic variation of CMV, together with mutation rates induced by a combination of short generation time and error-prone replication [6,14,15]. 

Isolates/variants of CMV can also pose as helper viruses to satellite RNAs (sat-RNAs), which are occasionally found accompanying the virus. These CMV sat-RNAs are small linear RNA molecules with sizes ranging from 332–405 nucleotides and are dependent on CMVs for replication, encapsidation and dispersion, but they are not necessary for the life cycle of the virus [16]. They can attenuate or exacerbate the symptoms induced by the helper viruses in specific plant hosts [17,18]. 

It is obvious that CMV epidemiology remains complex, as the virus symptomatology, rate of replication and transmission rate can change depending on the specific isolate, host plant species and presence of sat-RNA. Furthermore, under natural conditions, mixed infections by several viral species, strains or isolates/variants are quite common [19]. The importance of studying CMV epidemiology can be highlighted by the fact that there are no CMV-resistant varieties of tomatoes [20], and it was previously observed that resistance-breaking CMV variants continuously emerged in various resistant pepper cultivars [21]. A mixed infection incorporating different CMV strains is important from an evolutionary point of view, as reassortment and recombination are responsible for a share of CMV populational variation. Yet these events are quite rare, and their rates are host-dependent [14]. The rarity can be to some extent caused by the reduction of individual CMV strain numbers during transmission by aphids. This was demonstrated by Ali et al. (2006) [22], who used 12 restriction enzyme marker CMV mutants to determine the presence of a significant genetic bottleneck, as the number of CMV mutants was significantly reduced during the inoculation of new hosts by both *Myzus persicae* and *Aphis gossypii*. Even though reassortment and recombination events are relatively rare, new recombinant variants are being identified [15,23,24,25] and so are non-recombinant isolates with different biological and genetic properties [26,27,28,29,30]. 

The large number of combinations of CMV strains, satellite RNAs, plant hosts and coinfecting plant viruses can be overwhelming for analysis of CMV variation [14]. Accessing whole-genome data by next-generation sequencing methods is therefore crucial for addressing CMV molecular variation and the factors underlying its observed status, as it can provide sequence data for CMV and satellite RNAs present in samples as well as coinfecting viruses if the proper protocol is followed [31]. The genomic variation of CMV is important to analyze, as was demonstrated by a single amino acid substitution in the 1a protein of CMV which led to necrosis of *Arabidopsis thaliana* inoculated leaves, while not affecting CMV multiplication or systemic spread [32].

In this study, we analyzed the variability of the CMV genome based on 14 fully sequenced CMV variants infecting seven different host plant species in Western Slovakia, four of which contained sat-RNA. Defining new isolates/variants and addressing the genetic variability of CMV is necessary for understanding the underlying mechanisms during epidemic outbreaks and providing information required for precautionary measures in virus management.

## 2. Results

### 2.1. HTS Enabled to Identify CMV within Complex Viromes Reveals New CMV Hosts from the Papaveracea Family

High-throughput sequencing (HTS) of ribosomal-depleted total RNA revealed the presence of multiple virus species in 13 out of 14 plant samples, with only one sample having a single virus infection. Mapping of contigs and of individual HTS reads against reference genomes for each of the viruses identified in the de novo analysis enabled the nearly complete reconstitution of complete genomes of several potyviruses, i.e., papaya ringspot virus (PRSV), turnip mosaic virus (TuMV), watermelon mosaic virus (WMV) and zucchini yellow mosaic virus (ZYMV), a carlavirus (potato virus M (PVM)), a polerovirus (turnip yellows virus (TuYV)) and a deltapartitivirus (pepper cryptic virus-2 (PCV-2)). Moreover, in each analyzed sample, the nearly complete RNA1, RNA2 and RNA3 of CMV was found. Except for the sample LAS from greater celandine, in which CMV occurred as a single agent, all other samples presented double, triple or quadruple mixed virus infections (Table 1, Table S1). 

**Figure 1:**
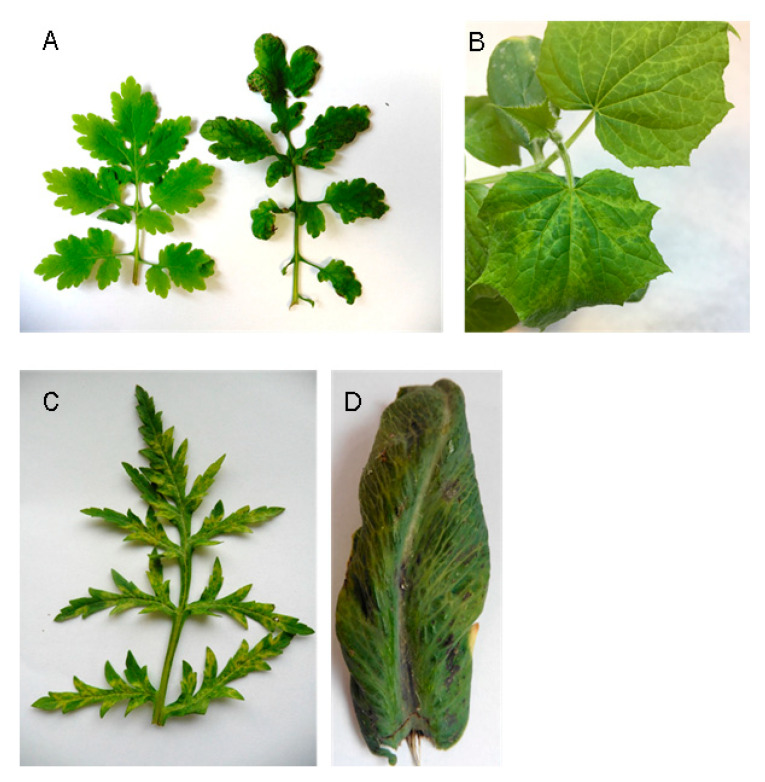
**The symptoms present in different original and experimental hosts**. (**A**) *Chelidonium majus* (healthy plant on the left, plant labelled as LAS on the right). (**B**) Experimental cucumber plant inoculated by CMV LAS. (**C**) *Papaver rhoeas* (sample PK2). (**D**) *Papaver somniferum* (sample PK1).

The presence of CMV was detected in leaf samples from common cucurbit (CP2, CS3, MVU2-21) or solanaceous hosts (MIH1, N65, SL50V, T1, T24, T50, T65, T101). Interestingly, three CMV hosts, previously unreported in Slovakia, were found, i.e., oilseed poppy (*Papaver somniferum* L., PK1), common poppy (*Papaver rhoeas* L., PK2) and greater celandine (*Chelidonium majus* L., LAS), all belonging to the *Papaveraceae* family (Table 1). Original PK1 and PK2 host plants displayed pronounced virus-like symptoms; however, due to the mixedness of the infections, they cannot be directly attributed to the respective viruses. A single CMV-infected greater celandine plant, from which the LAS isolate was isolated, displayed slight leaf deformations and puckering (Figure 1); however, CMV LAS -mechanically inoculated cucumber plants displayed systemic yellow mosaics on leaves.

### 2.2. Slovak CMV Isolates Belong to Two Different Genetic Groups

**Figure 2:**
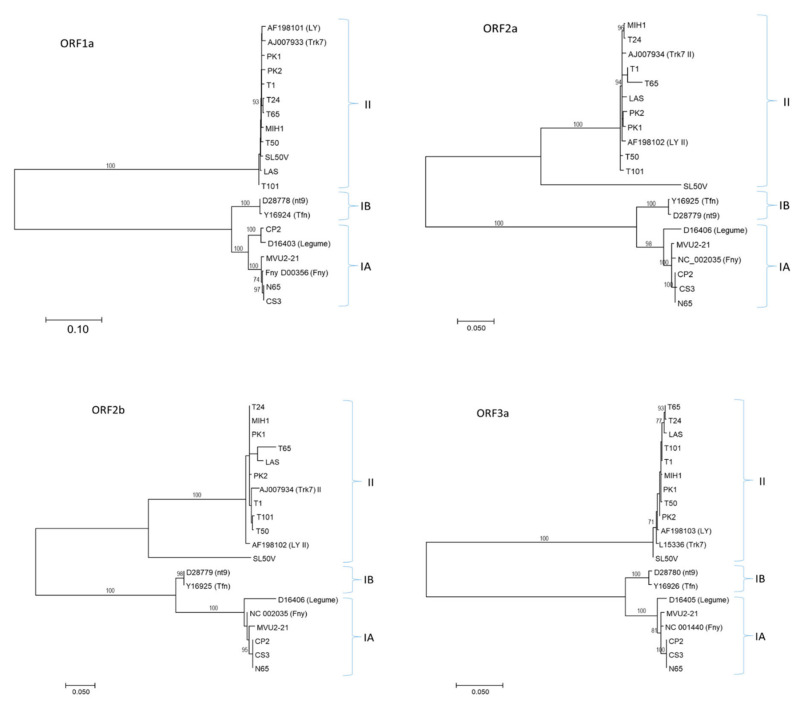
**Phylogenetic analyses of the complete coding sequences of RNA1 (ORF1a), RNA2 (ORF2a, ORF2b) and RNA3 (ORF3a) of CMV**. RNA3 ORF3b analysis presented the same tree topology as ORF3a. For each molecular group (IA, IB and II), two reference GenBank sequences are included, identified by their accession number and name. The scale bar indicates genetic distance. Bootstrap values higher than 70% (1000 bootstrap resamplings) are indicated. The phylogenetic analysis was inferred using maximum likelihood (ML) based on the Tamura–Nei model selected as the best-fit model of nucleotide substitution based on the Bayesian information criterion (BIC) as implemented in MEGA7.

The determined CMV RNA1, RNA2 and RNA3 genomic sequences showed typical cucumovirus organization. Based on the BLAST search and phylogenetic analysis, the Slovak CMV variants could be divided into two genetically different groups. While the variants CP2, CS3, MVU2-21 and N65 were genetically close to Group IA isolates, other variants (LAS, MIH-1, PK1, PK2, SL50V, T1, T24, T50, T65, T101) belonged to Group II (Figure 2). The CMV RNA nucleotide sequences reported in this paper have been deposited in the GenBank database under accession numbers listed in Table 1.

Obtained RNA1 nucleotide sequences encompassed the whole ORF 1a gene, although the coding sequences between variants of Group IA (2982 nt including stop codon) and Group II (2979 nt) slightly differed in length. This difference resulted in the deletion of one amino acid at position 552 of the protein. The mean distances at the amino acid level between Slovak variants of Group IA and Group II were low (0.6% and 0.3%, respectively), while the differences between the two groups reached 14.1%.

RNA2 contained two ORFs. ORF2a was 2523 nt and 2574 nt long for variants of Group II and IA, respectively, resulting in 17 aa longer translated products for Ia variants. The mean distance at the amino acid level between Slovak variants of Group Ia and Group II (except SL50V) were low (0.6% and 1.2%, respectively) and the differences between the two groups reached 24.4%. The ORF2 of the divergent SL50V variant was 2532 nt long. Although SL50V belonged to Group II, its deduced 2a product shared only ca. 82% identity in comparison with other Slovak variants from this group. The differences were randomly distributed along whole RNA2, without particular peaks of divergence. 

Similar to ORF2a, the length of partially overlapping ORF2b was found to be variable between Groups IA and II (303 and 333 nt, respectively, and 300 nt for SL50V) and deduced amino acid products were characterized by low mean intra-group divergence (0.5% and 2.0%). Again, the 2b protein of the SL50V variant displayed a low level of identity similar to other Slovak variants of Group II, reaching only 68.8%.

RNA3 contained two ORFs encoding the MP gene (840 nt) and the CP gene (657 nt) and revealed the lowest divergence among Slovak CMV variants, without indels for all characterized variants. Translated MP and CP products within both groups showed minimal divergence (0–0.2%), while the differences between the two groups reached 17.6% (MP) and 16.6% (CP).

Phylogenetic analysis of all Slovak variants with selected database sequences based on the respective coding regions showed a similar topology for the ML trees. As expected from the multiple alignments, divergent SL50V variants clustered in a separate branch within the Group II cluster in ORF2a and ORF2b. The BLAST search did not find a close relative to SL50V RNA2 or its deduced product, the closest being the Ack2 artichoke isolate from South Korea (LC487908) with 79.5% identity.

### 2.3. Satellite RNAs Were Found in Association with Some CMV Variants

**Figure 3:**
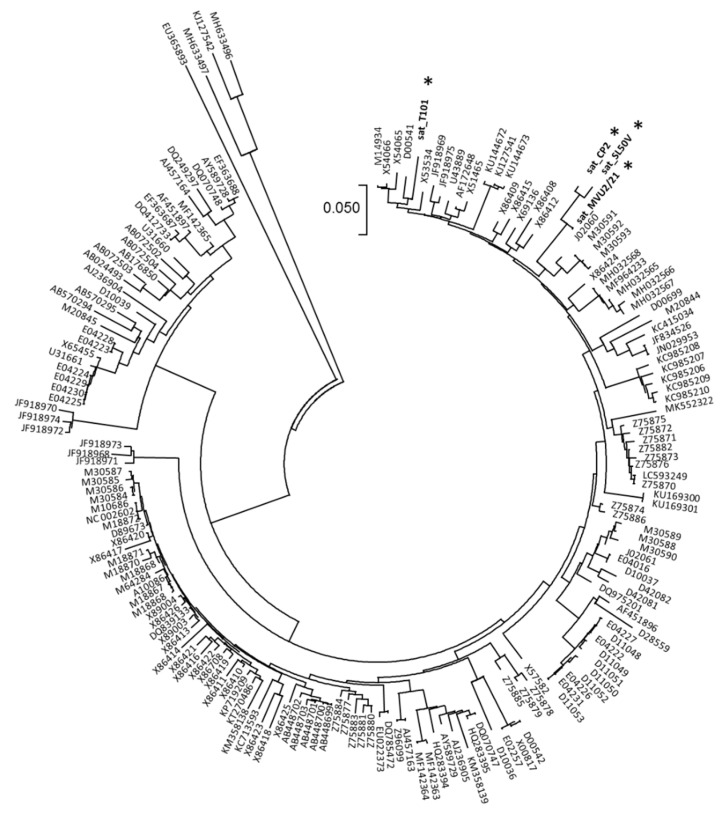
**Phylogenetic tree constructed from the aligned complete sequences of CMV sat-RNA**. The four Slovak sequences obtained in this work are marked by an asterisk. The phylogenetic analysis was inferred using neighbor joining based on the *p*-distance model implemented in MEGA7.

In four samples (T101, SL50V, CP2, MVU2-21) the presence of satellite CMV RNA was identified along with CMV. The mapping of HTS reads to reference sequences (NC_002602) resulted in CMV satellite genomes of 339 nucleotides (sat_MVU2-21), 392 nucleotides (sat_T101) and 393 nucleotides (sat_CP2, sat_ SL50V), respectively. The nucleotide identities to the reference NC_002602 sequence ranged from 70.1 to 79.7%. The phylogenetic analyses showed the genetic closeness of sat_CP2, sat_SL50V and sat_MVU2-21, clustering at the same phylogenetic branch, while sat_T101 clustered slightly away (Figure 3).

The association of satellite CMV RNA with the infection in all four original samples was further confirmed by RT-PCR using satCMV_skF/ satCMV_skR primers and Sanger sequencing of PCR amplicons.

Attempts were made to test the mechanical transmissibility of two satellite CMVs from original plant samples (tomato for SL50V and squash for CP2) to healthy cucumber plants. In both cases, 100% transmissibility was achieved for CMV and satellite CMV, as tested by RT-PCR. Furthermore, the transmission of all coinfecting viruses present in the original SL50V sample (PVY) and CP2 (PRSV, ZYMV, WMV) was noted. The SL50V-inoculated cucumber plants displayed mild leaf yellowing, while those inoculated with CP2 displayed pronounced leaf mosaics, yellowing and dwarfing. However, as both samples were co-infected by additional viruses, the roles of CMV and the satellite CMV in the observed symptomatology cannot be linked. 

## 3. Discussion

CMV is known to infect the widest range of host plants, including more than 1300 species from more than 100 taxonomic families [8,29], including vegetable crops, ornamental, medicinal plants and weeds, while the number of known hosts is still increasing [33] and cross-kingdom viral infection by CMV was documented in a phytogenic fungus [34]. The wide host range of CMV is mostly associated with its ability to be transmitted by over 80 aphid species [12], including *Myzus persicae* and *Aphis gossypii*. The severity of CMV outbreak can be very high in vegetable crop fields growing introduced crop varieties and is common because of their high productivity, as was previously reported during several CMV outbreaks in the Mediterranean Basin [35]. CMV is spread worldwide and in addition to vegetable crops, it can also infect ornamental plants, medicinal plants and weeds [29].

In this work, CMV was identified in several cucurbit or solanaceous hosts, known as common hosts of this pathogen. On the other hand, three unusual CMV hosts (not yet reported from Slovakia) were found, i.e., oilseed poppy (PK1), common poppy (PK2) and greater celandine (LAS), all belonging to the *Papaveraceae* family (Table 1). Greater celandine (*Ch. majus*) is a perennial herbaceous yellow-flowering plant of woodland rides, hedgerows and roadsides. Common poppy (*P. rhoeas*) is a cosmopolitan red-flowering annual plant. Both species are commonly found in/near fields or gardens in Slovakia, as weeds. On the contrary, oilseed poppy (*P. somniferum*) is grown in Slovakia as an agricultural crop to produce edible seeds. To our knowledge, this is the first report of CMV infecting greater celandine. Information on CMV naturally infecting common and oilseed poppy is scarce and there is a lack of experimental and sequence data. A report of CMV host range from Japan included common poppy as a natural host of CMV [36], while concern was expressed over possible CMV infection of oilseed poppy, as for all *Papaver* spp., by Kubelková and Špak [37]. Our results thus further confirm the broad host range of CMV [8,29,33,38]. Knowledge of potential wild plant virus hosts is important for virus management practices, as they constitute an important part of the agroecological interface [39]. For example, common poppy (from the *Papaveraceae* family) is frequently grown within or in close proximity to oilseed poppy fields [40] and it is therefore expected that CMV could be transmitted between the two species.

Obtained genomic data have confirmed the presence in Slovakia of CMV variants belonging to Groups IA and II, similar to a recent study in Poland, a neighboring country with similar climatic conditions, where the prevalence of Group II variants was noted [24]. This is in accordance with previous studies, where these groups were considered to be spread worldwide, as opposed to Group IB, which is mostly restricted to Asia [8], although some studies from Europe reported the occurrence of the IB group in the Mediterranean area [18,41]. 

Individual genes showed different levels of variation within and between molecular groups. Within-group divergence of Slovak variants in this study ranged from 0–0.2% (for ORFs encoding CP and MP on RNA3) to 0.5–2.0% (for ORF2b on RNA2). Low regional genetic diversity of CMV was previously observed [23]. Variability between groups of Slovak CMVs was much larger compared to within-group variation, as was expected due to large nucleotide diversity between groups previously described [8]. Based on our data, it would also seem that RNA2 and ORFs present there show the highest levels of variability, both within and among individual groups. Similar results were reported previously [23,42]. It is obvious that the CMV population in Slovakia possesses a certain level of variability which should be accounted for during diagnostic testing [43]. 

HTS enables the determination of millions of sequence reads in a short time and this has opened novel opportunities for the diagnosis of the plant virome without prior information about pathogenic properties [43]. There is also growing evidence supported by previous HTS studies that complex infection of plants by several viruses is the rule rather than the exception, and therefore interaction between viruses needs to be addressed in plant virus management strategies [44]. These data are also confirmed by our study, where only one plant sample (LAS) was characterized by single virus infection. This situation also makes it difficult to attribute a specific symptom to CMV infection, taking into account the potential synergistic/antagonistic effects of some virus combinations for virus etiology. A paper focused on viral relationships in mixed infection provided evidence for CMV and Southern tomato virus (STV) double-infection synergy, as CMV titers increased in mixed infection and so did symptoms during early infection [20].

Moreover, in previous works analyzing viral populations in Slovakia, mixed infections involving different variants of the same virus species were noted, both for annual (PVY/tomato [45]) or perennial hosts (CVA/cherry [46]; GRSPaV/grapevine [47]). In our experiments, only a single-variant CMV population was detected in each sample (as supported by analysis of de novo contig assemblies and visual inspection of reads), enabling the accurate reconstruction of nearly full-length CMV genomes. 

The widely used standard molecular diagnostic tests targeting virus pathogens (based on the use of specific oligonucleotides or probes) require knowledge about their genomic properties [43]. Especially for newly described viruses or highly diverse strains of known viruses, such knowledge can be biased by insufficient numbers of genomic data or their restriction to some geographical area or host. Our study underlines the necessity of proper diagnostic methods for research and detection purposes, as well-known and widely distributed viruses, such as CMV, can still hide a part of their diversity, as documented here and previously by several papers describing unusual CMV mutants, recombinants or reassortants [15,25,42], such that there is a need for continued study of viral diversity. Indeed, because of the robustness of the HTS method, we were able to address the variation of CMV isolates in our samples and detect a potential naturally occurring reassortant CMV strain in tomato (SL50V).

Satellites of CMV can often modulate the symptomatic response of a host and the replication rates of helper viruses [48]. Symptomatic changes can be attenuated, exacerbated or remain the same in the presence of CMV sat-RNA [17,18]. This makes the presence of satellite RNAs problematic for objective symptomatic screening. Sat-RNAs of CMV were detected in four samples in this study, comprising three different plants of common solanaceous and cucurbitaceous hosts. The size of the CMV sat-RNA determined in this work (339–393 nts) is in the range of available CMV sat-RNA sequences in GenBank (*n* = 177, size 307–405 nts; https://www.ncbi.nlm.nih.gov/nuccore/, accessed on 8 November 2021). 

The occurrence of CMV sat-RNA is considered generally low and the symptoms exacerbating CMV sat-RNA are rarest, while symptom attenuation is the most common effect of CMV sat-RNA observed [8]. Symptom attenuation can be expected, as the sat-RNA and helper virus compete for limited virus and host resources for replication [49]. It was also proven that cross-protection can be used using stand-alone non-necrogenic CMV sat-RNA [50], without its helper virus, to prevent severe symptom development after secondary infection by a necrogenic sat-RNA including a CMV strain. On the other hand, some CMV sat-RNAs were previously reported to cause necrogenic symptoms in plants, resulting in large economical losses [18]. Therefore, the presence of sat-RNAs is important to consider as potential enhancers of symptom expression in order to address and assign symptoms to the proper causative agents, even more so in cases of mixed infection. This is supported by a study where the addition of sat-RNAs to CMV single infection reduced the accumulation of CMV, while in mixed infections with ZYMV and WMS the suppressive effect of CMV sat-RNA on its helper virus was reversed and sat-RNA accumulation increased [51]. 

In terms of genetic variability, sat-RNAs are much more diverse when compared with other RNA genomes, which could be accounted for by lower negative selection, as the sat-RNA itself is not vital for the virus [8]. Still, a partially conserved structure was reported for CMV sat-RNA-mediated inhibition of CMV accumulation, specifically inhibition of RNA1 and RNA2 accumulation by biologically relevant structures within the sat-RNA genome, predicted to be present in 70% of CMV sat-RNAs [49].

## 4. Materials and Methods

### 4.1. Samples and HTS Analysis

All the samples consisted of one fully developed leaf from an actively growing part of the plant collected during the vegetation period (July to September) between 2017–2021. 

Total RNAs were extracted from leaves (ca 0.2 g) using a Spectrum Plant Total RNA Kit (Sigma Aldrich, St. Louis, MO, USA). To enrich the viral fraction, ribosomal RNAs were removed from the total RNAs using the Ribo-Zero rRNA Removal Kit (Illumina, San Diego, CA, USA). The samples of ribosomal-depleted total RNAs were used for double-stranded cDNA synthesis using the SuperScript II kit (Thermo Fisher Scientific, Waltham, MA, USA). The cDNA was then purified with the 2.2 x AMPure XP beads (Beckman Coulter, Indianapolis, IN, USA) and quantified with the Qubit 2.0 Fluorometer (Thermo Fisher Scientific, Waltham, MA, USA). The samples were then processed with the transposon-based chemistry library preparation kit (Nextera XT, Illumina, San Diego, CA, USA). Low-cycle PCR and mutual indexing of the fragments was carried out. Fragments were purified with 1.8 x AMPure XP beads (Beckman Coulter, Indianapolis, IN, USA) without size selection. Fragment size structure of the DNA libraries was assessed using the Agilent 2100 Bioanalyzer (Agilent Technologies, Santa Clara, CA, USA). The equimolar pool of 4nM DNA libraries was denatured, diluted to 10 pM and sequenced (300 bp paired-end sequencing) on the Illumina MiSeq platform (Illumina, San Diego, CA, USA).

Bioinformatic analysis of the obtained high-quality trimmed reads was carried out using different strategies. First, de novo contig assembly was performed with the Geneious Prime 2020 (Biomatters Ltd., Auckland, New Zealand) and CLC Genomics Workbench 10.1.1 (CLC bio, Aarhus, Denmark) softwares. The resulting contigs were BLASTed (Blastn, BlastX) against the nucleotide database at NCBI (https://blast.ncbi.nlm.nih.gov/Blast.cgi accessed on 22 November 2021). Each resulting viral contig from the previous step was used as a reference in an iterative procedure to validate or extend the contig using the Geneious Prime 2020 software with different custom mapping options. 

The obtained nearly complete CMV genomes of RNA1, RNA2 and RNA3 were compared with the CMV sequences representative of Groups IA, IB and II retrieved from the GenBank database (www.ncbi.nlm.nih.gov, accessed on 22 November 2021).

Phylogenetic analyses and comparisons were performed using the MEGA v.7 [52] and DnaSP v.5 [53] programs.

### 4.2. Mechanical Transmission and Detection of Satellite CMV

Inoculum was prepared from original leaf samples by grinding the leaves stored at −80 °C in Norit buffer (1/10, *w*/*v*) (https://www.dsmz.de/fileadmin/_migrated/content_uploads/Inoculation_01.pdf, accessed on 22 November 2021). The cucumber plants (cv. Vanda) at the cotyledon stage per variant were mechanically inoculated by rubbing the surface of the cotyledons previously dusted by carborundum. Ten cucumber plants in two independent experiments were inoculated for each virus sample. 

The presence of satellite CMV was tested in the cucumber leaves 14 days post-inoculation by RT-PCR using the newly designed primer pair satCMV_skF (5′-GGTTATATCTACGTGAGGATC-3′)/satCMV_skR (5′-ACCACCTAACAGAGTGTTTC-3′) designed from the HTS-based sequences obtained, generating a fragment of 278 bp. The accuracy of PCR amplicons was verified by Sanger sequencing of PCR products using the same primers. The presence of CMV and other viruses (Table 1) was checked by DAS-ELISA using commercial antibodies (Bioreba AG, Reinach, Switzerland).

## 5. Conclusions

HTS technology has brought new possibilities to characterize the complexity of the plant virome, as confirmed by multiple infections found in most of the CMV-infected samples analyzed in this work. This fact needs to be considered in plant virus management strategies due to possible antagonistic/synergistic interactions between viruses in complex infection. Moreover, sat-RNAs were detected and associated with four CMV variants belonging to Groups IA and II. Although the role of CMV sat-RNA in disease etiology could not be determined due to the mixed infection, in a general way their presence cannot be underestimated as they may have profound effects on symptom development, ranging from lethal necrosis to disease attenuation. 

CMV is a cosmopolitan virus infecting a large spectrum of mono- and dicotyledonous hosts. We have shown that three *Papaveraceae* members can act as natural CMV hosts. Besides the direct threat to a culture crop (oilseed poppy), CMV was found in two weed species (common poppy and greater celandine) commonly present in the vicinity or within agricultural crops in many parts of the world. Therefore, both species may pose as virus reservoirs and contribute to the vector-mediated spread of the virus.

## Figures and Tables

**Table 1:** List of CMV isolates analyzed in this study and host characteristics.

Isolate	Original Host	Locality, Year of Sampling	GenBank Accession Number	Presence of CMV Satellite (GenBank Accession Number)	Other Viruses Identified by HTS
CP2	*Cucurbita pepo*	Pezinok, 2019	MZ298665 ^a^, MZ298675 ^b^, MZ298685 ^c^	Yes (ON493796)	PRSV, ZYMV, WMV
CS3	*Cucumis sativum*	Cífer, 2018	MZ298666, MZ298676, MZ298686	No	ZYMV, WMV
LAS	*Chelidonium majus*	Bratislava, 2017	MZ298667, MZ298677, MZ298687	No	None
MIH1	*Capsicum annum*	Čachtice, 2017	MZ298668, MZ298678, MZ298688	No	PCV-2
MVU2-21	*Cucumis melo*	Velke Ulany, 2021	ON409882, ON409886, ON493791	Yes (ON493795)	WMV, ZYMV, CmEV
N65	*Capsicum annum*	Čachtice, 2017	ON409883, ON409887, ON493792	No	WMV, PCV-2, BPEV
PK1	*Papaver somniferum*	Pezinok, 2017	MN792886, MN792887, MN792888	No	TuMV
PK2	*Papaver rhoeas*	Pezinok, 2017	ON409881, ON409885, ON493790	No	TuMV, TuYV
SL50V	*Solanum lycopersicum*	Pezinok, 2018	MZ298669, MZ298679, MZ298689	Yes (ON493794)	PVY
T1	*Solanum lycopersicum*	Paňa, 2017	MZ298671, MZ298681, MZ298691	No	PVY
T24	*Solanum lycopersicum*	Nitra, 2017	MZ298672, MZ298682, MZ298692	No	PVY
T50	*Solanum lycopersicum*	Plavecký Mikuláš, 2017	MZ298673, MZ298683, MZ298693	No	PVM, PVY
T65	*Solanum lycopersicum*	Sološnica, 2017	MZ298674, MZ298684, MZ298694	No	PVM, PVY
T101	*Solanum lycopersicum*	Pezinok, 2019	MZ298670, MZ298680, MZ298690	Yes (ON493793)	PVY

^a^ RNA1. ^b^ RNA2. ^c^ RNA3.

## Data Availability

The nucleotide sequences reported in this paper are deposited in the GenBank database (www.ncbi.nlm.nih.gov) under the accession numbers listed in the text.

## Supplementary Materials

The following supporting information can be downloaded at: https://www.mdpi.com/article/10.3390/plants11131665/s1, Table S1: Analysis of obtained HTS data in relation to CMV.
