# Supplementary material for: High-Throughput Sequencing Discloses the Cucumber Mosaic Virus (CMV) Diversity in Slovakia and Reveals New Hosts of CMV from the Papaveraceae Family
Source: Plants (Basel). 2022 Jun 23;11(13):1665. doi: 10.3390/plants11131665 (PMC9269241; doi:10.3390/plants11131665)
Supplement: Supplementary file 1 [file plants-11-01665-s001.zip › TableS1.pdf]

Table S1. Analysis of obtained HTS data in relation to CMV

isolate		total number of reads /mean lenght	number of reads mapped to the determined CMV sequence	average depth of coverage
CP2	RNA1	5690694/153.2	300397	4012.7
	RNA2		281196	5796.6
	RNA3		850791	53496
CS3	RNA1	5662550/144.6	69741	2292.2
	RNA2		67140	2561.7
	RNA3		114354	62355.9
LAS	RNA1	2302978/82.7	135202	3117.2
	RNA2		123599	3004.3
	RNA3		215450	7632.1
MIH1	RNA1	3732476/115.3	10770	279.1
	RNA2		9041	234.5
	RNA3		19661	937.8
MVU2-21	RNA1	43583778/130.7	2236858	82278
	RNA2		2337838	94225
	RNA3		7933097	434477
N65	RNA1	5511704/143.3	2997	116.4
	RNA2		2054	99.8
	RNA3		5089	277.6
PK1	RNA1	3770865/122.1	16033	4837
	RNA2		12367	3922.4
	RNA3		19797	8853.6
PK2	RNA1	3901516/148.5	275071	11309.8
	RNA2		530240	20180.9
	RNA3		638081	31380.8
SL50V	RNA1	2550640/119.4	36714	1262
	RNA2		30983	1106.5
	RNA3		76734	4279.8
T1	RNA1	1855568/115.3	597167	19887.7
	RNA2		511161	18570.9
	RNA3		589683	3733.3
T24	RNA1	2215328/116.7	514614	15306.6
	RNA2		313832	11865.1
	RNA3		815834	44192.8
T50	RNA1	2466092/117.0	73536	1993.6
	RNA2		76587	2443.1
	RNA3		83607	4552.9
T65	RNA1	2886468/122.1	54020	5987.3
	RNA2		36774	3243.8
	RNA3		63670	7655.1
T101	RNA1	3168840/176.4	89645	3176.9
	RNA2		72750	2936.9
	RNA3		168866	10362.1
